# Genome Sequences of Cluster G Mycobacteriophages Cambiare, FlagStaff, and MOOREtheMARYer

**DOI:** 10.1128/genomeA.00595-15

**Published:** 2015-06-18

**Authors:** Welkin H. Pope, David A. Augustine, Daniel C. Carroll, Julia C. Duncan, Kyle M. Harwi, Rachel Howry, Bhavita Jagessar, Brandon A. Lum, Justin W. Meinert, Jeffrey S. Migliozzi, Katherine A. Milliken, Chandler J. Mitchell, Akanksha S. Nalatwad, Kaila C. Orlandini, Michael J. Rhein, Vishmayaa Saravanan, Brett A. Seese, Johnathon G. Schiebel, Kayla B. Thomas, Nancy L. Adkins, Karen L. Cohen, Varun B. Iyengar, Hannah Kim, Zachary J. Kramer, Matthew T. Montgomery, Claire E. Schafer, Kellyn E. Wilkes, Sarah R. Grubb, Marcie H. Warner, Charles A. Bowman, Daniel A. Russell, Graham F. Hatfull

**Affiliations:** Department of Biological Sciences, University of Pittsburgh, Pittsburgh, Pennsylvania, USA

## Abstract

Mycobacteriophages Cambiare, FlagStaff, and MOOREtheMARYer are newly isolated phages of *Mycobacterium smegmatis* mc^2^ 155 recovered from soil samples in Pittsburgh, PA. All three genomes are closely related to cluster G mycobacteriophages but differ sufficiently in nucleotide sequence and gene content to warrant division of cluster G into several subclusters.

## GENOME ANNOUNCEMENT

Mycobacteriophages—viruses infecting mycobacterial hosts such as *Mycobacterium smegmatis* and *Mycobacterium tuberculosis*—have proven to be informative about viral diversity and evolution, as well as providing tools for understanding tuberculosis (1, 2). Bacteriophages have been effective models for elucidating fundamental concepts in molecular biology and continue to reveal novel aspects of gene control (3).

Mycobacteriophages Cambiare, FlagStaff, and MOOREtheMARYer were isolated from soil samples from Pittsburgh, PA, purified, amplified, and shown to have similar siphoviral morphologies. DNA was isolated from each and sequenced using an Illumina MiSeq with 140-bp single-end reads. Reads from each project were assembled using Newbler, and contigs were generated with 1,365-, 4,457-, and 471-fold coverage for Cambiare, FlagStaff, and MOOREtheMARYer, respectively. The genomes are 45,161, 44,476, and 44,492 bp long, respectively, and all three have 10-bp 3′ extensions with the sequence 5′-CCCATGGCAT, evident from the coverage profile of the assembly. Protein coding sequences were predicted using Glimmer and GeneMark using both heuristic and *M. smegmatis* models, and 67, 65, and 67 putative genes were identified in Cambiare, FlagStaff, and MOOREtheMARYer, respectively. None of the genomes encode tRNAs.

BLASTN shows that all three genomes are related at the nucleotide sequence level to G cluster phages, which with inclusion of the three new genomes contains 20 phage members in GenBank (4–6). Curiously, Cambiare, FlagStaff, and MOOREtheMARYer are more distantly related to cluster G phages such as BPs, Angel, Hope, and Halo (4) than those are to themselves, suggesting that the G cluster warrants division into subclusters. Comparison of average nucleotide identities (ANI) supports four subclusters, G1, G2, G3, and G4. Cluster G1 contains phages Angel, Avrafan, Chy2, Chy3, Clark, DNAIII, Bo4, BPs, Gomashi, Guo1, Halo, Hope, Legendre, Leo, Liefie, and Sedge. Subcluster G2 contains Cambiare and FlagStaff, and phages MOOREtheMARYer and Jolie2 are the sole members of subclusters G3 and G4, respectively.

Cambiare, FlagStaff, and MOOREtheMARYer and the other subcluster G2, G3, and G4 phages are architecturally distinct from other cluster G phages in that they lack the centrally located immunity cassette (integrase and repressor) required for integration-dependent immunity (3, 7). They carry the genes immediately to the left and right of the immunity cassette, indicating a loss of these functions without replacement. There are also notable differences at the right end of the virion structure and assembly operon, within a set of tail genes in Cambiare, FlagStaff, and MOOREtheMARYer that is different from the set in the subcluster G1 phages. Specifically, BPs (subcluster G1) gene *22*—which has previously been shown to be the site of mutations giving rise to an expanded host range (8)—is substituted with a different tail gene, along with 2 to 4 additional genes presumably also involved in tail structure. These variations suggest that Cambiare, FlagStaff, and MOOREtheMARYer may have host preferences different from those of the other cluster G phages. We note that Cambiare, FlagStaff, and MOOREtheMARYer also lack the MPME elements present in several other cluster G phages.

### Nucleotide sequence accession numbers. 

The Cambiare, FlagStaff, and MOOREtheMARYer genome sequences were submitted to GenBank under the accession numbers KR080198, KR080197, and KR080202.
